# Benefit of intrapleural fibrinolytic therapy in the treatment of complicated parapneumonic effusion and empyema

**DOI:** 10.11604/pamj.2024.47.54.15439

**Published:** 2024-02-08

**Authors:** Takoua Merhabene, Souheil Zayet, Amira Jamoussi, Samia Ayed, Salwa Mansouri, Jalila Ben Khelil, Mohamed Besbes

**Affiliations:** 1 Abderrahmen Mami Hospital, Intensive Care Unit, Tunis, Tunisia,; 2 Université Tunis El Manar, Faculté de Médecine, Tunis, Tunisie

**Keywords:** Empyema, microbiology, chest ultrasound, antibiotics, fibrinolysis

## Abstract

Our study aimed to assess the benefit of intrapleural fibrinolysis before resorting to surgery to treat complicated parapneumonic effusion and empyema. We conducted a retrospective and descriptive study, including all patients hospitalized in the intensive care unit (ICU) of the Abderhaman Mami hospital, Tunisia for empyema treated with instillation of intrapleural fibrinolytic therapy between the 1^st^ January 2000 and 31^st^ December 2016. In all patients, empyema was diagnosed on clinical features, imaging findings (chest X-ray, thoracic echography and/or computed tomography (CT), and microbiological data. The fibrinolytic agent used was streptokinase. The efficiency of intrapleural fibrinolytic therapy was judged on clinical and paraclinical results. Among 103 cases of complicated parapneumonic effusion and empyema, 34 patients were included. The mean age was 34 years [15-81] with a male predominance (sex ratio at 2.77). Median APACH II score was 9. Fifty (50%) of the patients (n=17) had no past medical history; addictive behavior was described in 17 patients (50%). All patients were admitted for acute respiratory failure and one patient for septic shock. Pleural effusion was bilateral in 7 patients. Bacteria isolated were Streptococcus pneumonia (6 cases), Staphylococcus aureus (3 cases, including one which methicillin-resistant), Staphylococcus epidermidis (1 case), anaerobes (5 cases), and Klebsiella pneumoniae (1 case). First-line antimicrobial drug therapy was amoxicillin-clavulanate in 20 patients. A chest drain was placed in all cases in the first 38 hours of ICU admission. The median number of fibrinolysis sessions was 4 [2-9] and the median term of drainage was 7 days [3-16]. No side effects were observed. Video-assisted thoracoscopic surgery was proposed in 5 patients. The median length of hospitalization stay was 15 days [6-31]. One patient died due to multi-organ failure.

## Introduction

Pleural infections represent an important group of disorders characterized by the invasion of the pleural space by pathogens which may progress to a frank empyema [1]. Pleural effusion (PE) occurred in 36% to 57% of patients hospitalized with pneumonia. In these patients, 10% may develop empyema or complicated parapneumonic effusion (CPPE), which is secondary and associated with 14% to 20% of cases to fatal prognosis [1]. There are several classifications of CPPE based on secondary evolution (exudative, fibrino-proliferative with evidence of loculation, and development of fibrous peel) and fiuid characteristics (clear, viscous, or pus). Chest tube drainage (CTD) associated with adequate antimicrobial drug therapy is usually sufficient to treat uncomplicated PE. In these cases, chest drainage represents the most effective and prompt therapy for healing. However, it is often insufficient when the effusion is characterized by an increased viscosity and loculation [2]. Limited evidence exists to guide clinicians in selecting the ideal drainage technique compatible with each situation because of the broad variation in intrapleural infection's extent, presence of locules, comorbidities, respiratory status, and underlying pathogen's virulence. Many randomized controlled trials (RCTs) suggested that fiushing the pleural space with a fibrinolytic agent such as streptokinase or urokinase may help in breaking down the fibrinous bands or loculations which leads to total drainage of infected pleural fluid by increasing significantly the amount of pus drained. Meta-analysis of these RCTs showed that intrapleural fibrinolytic (IPF) therapy confers a benefit in reducing the requirement for surgical interventions.

## Methods

**Objectives:** we conducted this study to demonstrate the benefit of adding IPF therapy to intercostal CTD in the treatment of CPPE and empyema by reducing either morbid mortality or the need for subsequent surgical debridement of the pleural space.

**Design:** this was a retrospective and descriptive study, including all patients hospitalized in the intensive care unit (ICU) of Abderhaman Mami Hospital, Tunisia for CPPE or empyema between 1^st^ January 2000 and 31^st^ December 2016 with instillation of IPF. Empyema was diagnosed on clinical presentation, chest X-ray, thoracic echography, and/or computed tomography and microbiological data. The fibrinolytic agent used was streptokinase. It was administrated daily at a dose of 250,000 IU diluted in 250 ml of normal saline instilled in the pleural cavity through the CTD. Then, the chest drain was clamped for 2 hours, during this period we recommended that patients frequently change their position (right and left position), then the chest tube was reconnected to aspiration. We performed in all patients a thoracic ultrasound daily to control the PE. Further instillations were stopped after ultrasound follow-up if severe complication occurred and/or if drained fluid through the tube was < 100 cc in 24 hours provided that the tube was patent and properly positioned. Otherwise, we planned to continue the daily instillation as long as the drained fluid volume is > 100 cc with a maximum of 14 doses. According to Maskell *et al*. [3], the efficiency of IPF therapy was judged on clinical and para-clinical data.

## Results

**Epidemiological characteristics:** during the study, 103 cases of CPPE and empyema were initially hospitalized in the ICU. Only 34 patients were included (incidence rate of 33% and overall incidence of 4 per 1000 admissions). Median age was 34.5 years [15-18] with a male predominance (sex-ratio=2.77). Underlying comorbidities were present in 47% of patients (Table 1). All CPPEs were community-acquired.

**Table 1:** epidemiological characteristics

Characteristics in studied patients (n=34)		Value
Age (years), (average, extremes)		34.5 [15-81]
Comorbidities (Number, %)	All cases	16 (47)
	Diabetes mellitus	7 (20.5)
	Heart disease	6 (17.6)
	Thoracic trauma	2 (5.9)
	Hepatitis	2 (5.9)
	Chronic obstructive pulmonary disease	1 (2.9)
Addiction (Number, %)	All cases	17 (50)
	Tobacco	13 (38.2)
	Tobacco and alcohol	3 (8.8)
	Tobacco, alcohol and drug-addiction	1 (2.9)

**Clinical diagnosis:** all patients were admitted for acute respiratory failure; the mean APACH II score was 9. Signs of pleural infection were mostly present in addition to clinical symptoms of pneumonia: fever, cough, sputum production, dyspnea, and pleuritic chest pain. Only one patient has septic shock at admission. Reduced tactile fremitus was the most common sign in our population. The main functional and physical signs are illustrated in (Table 2).

**Table 2:** functional symptoms and descriptive physical examination at admission

Functional signs (n=34)	Number (%)
Fever	28 (82.35)
Chest pain	24 (70.58)
Productive cough	19 (55.88)
Dyspnea	34 (100%)
Septic shock	1 (2.94%)
**Physical signs**	**Number (%)**
Reduced tactile fremitus	34 (100)
Temperature <36°C or > 38°C	28 (82.35)
Bronchial or diminished breath sounds	25 (73.52)
Heart rate > 100 beats / min	23 (67.64)
Respiratory frequency > 30 cycles / min	21 (61.76)
Mean Blood pressure (mmHg)	80 ± 11.43

### Paraclinic characteristics

**Figure 1:**
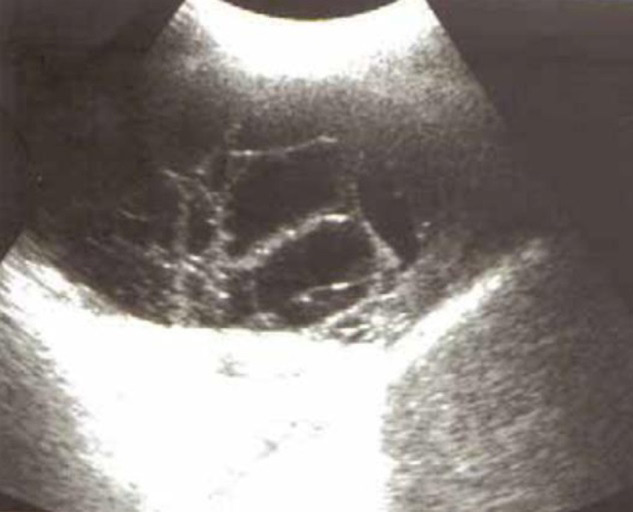
multiples septations within a parapneumonic effusion

All patients had abnormal arterial blood gases: 41.2% (n=14) are hypoxemic with an average PaO2 of 64 ± 21 mmHg. Metabolic acidosis (HCO3- < 20 mmol/l) was observed in 7 patients (Table 3). All patients had inflammatory syndrome (white blood count (WBC) average was 22000/mm^3^ [7400-39000]). The biological data are also illustrated in Table 3. At admission, all patients had a chest X-ray and transthoracic ultrasonography (TUS) (Figure 1). Seven patients (20.6%) had bilateral PE. The most common echographic appearance was hyperechoic in 20 cases. Chest CT was performed in 19 patients (55.9%). It showed an isolated PE in 15 cases and an associated pneumonia in 4 cases. Control ultrasonography was performed daily for all patients to check the vacuity of the pleura. On pleural aspiration, the fluid was cloudy in 58.8% of cases and sero-fibrinous in 29.4%. In the remaining cases, the fluid was either clear or sero-hematic. In all cases, pleural fluid analysis showed an exudative liquid, Rivalta positive. The mean intra-pleural WBC was 7013 cells/mm3 [140-24000]. The most prevalent bacteria isolated in our study were *Streptococcus pneumoniae* in 6 cases followed by anaerobes in 5 cases and *Staphylococci aureus* in 3 cases (one was methicillin-resistant (MRSA)) (Table 4).

**Table 3:** biological parameters

Venous blood gases	
**Parameters (n=34)**	**mean±DS**
pH	7.41 ± 0.06
PaO2 (mmHg)	64 ± 21
paCO2 (mmHg)	30.4 ± 10
HCO3- (mmol/L)	24.3 ± 5.7
SaO2 (%)	93.6 ± 2.7
**Biological parameters**	
**Biological data (n=34)**	**mean±DS**
Urea (mmol/L)	8.5 ± 8.9
Creatinine (umol/L)	139.8 ± 104
Na+(mmol /L)	133 ± 5
K+ (mmol /L)	3.7 ± 0.6
Prothrombin time (%)	74 ± 24
Lactates (mmol/L)	3,7 ± 1.46
Hematocrit (%)	36.4 ± 7
Hemoglobin (g/dl)	12 ± 2.4
WBC *103 (mm3)	22 ± 8.2
Platelet*103(mm3)	375 ± 154
C-reactive protein (mg/L)	290 ± 26.56

**Table 4:** distribution of isolated bacteria

Organisms cultured organisms cultured in pleural infections (n=34)	Number (%)
*Streptococcus pneumonia*	6 (17.64)
Anaerobes	5 (14.7)
*Staphylococcus aureus*	3 (8.82)
*Staphylococcus epidermidis*	1 (2.94)
*Klebsiella pneumoniae*	1 (2.94)
Serratia marcescens + Pseudomonas aeruginosa	1 (2.94)
Negative culture	17 (50)

### Treatment

**Figure 2:**
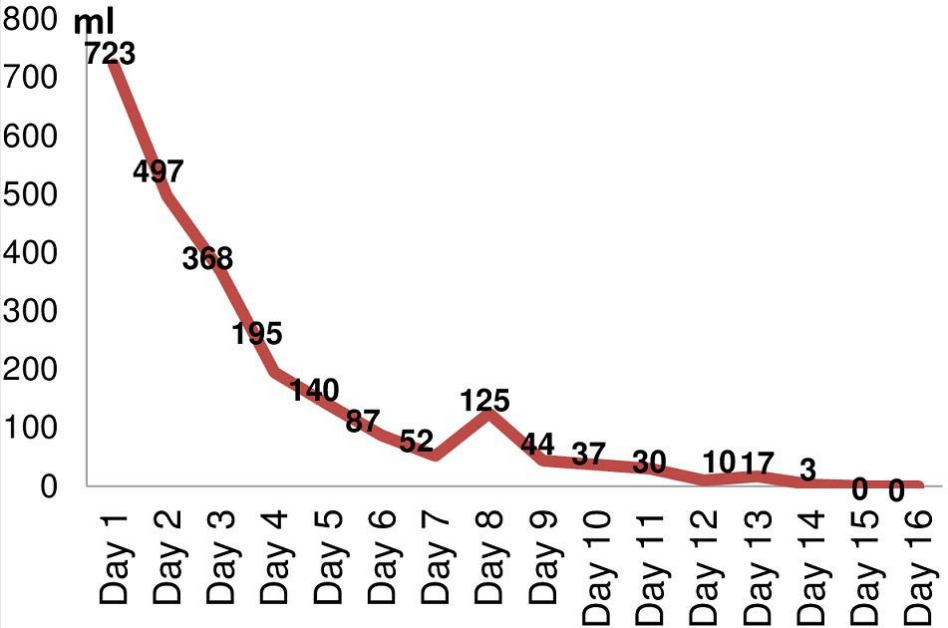
average of fluid volume per day after instillation of intrapleural fibrinolytic

All patients had initially closed intercostal chest drainage with a size 32 French. The chest tube was placed under the water seal system. The first dose of fibrinolytic therapy had been started in the first 38 hours after CTD placement. Instillation was repeated as long as no serious complication occurred. The median drained fluid volume was 2450 ml [1400-4500] after a maximum of 9 doses. The median number of instillation sessions of fibrinolysis was 4 and the mean duration of drainage was 7 days (Table 5). The most important drained fluid volume was on Day 1 with an average of 723 ml [400-1500] (Figure 2). All patients with CPPE have received intravenous antibiotics. Initial antibiotic coverage was dictated by guidelines treatment of community-acquired CPPE and managed according to blood and pleural fluid microbial cultures. Amoxicillin-clavulanate was initially used in 53% of cases. The remainder of the prescribed antibiotic therapy is detailed in Table 6. After antimicrobial susceptibility testing, the same molecule was maintained in 50% of cases; it was simplified in 30% and enlarged to cover the isolated germ in 20%. The mean duration of antimicrobial drug therapy was 15.3 days [7-30]. The use of mechanical ventilation and vasopressors was necessary in only one case. In addition, there was no need for extra renal replacement therapy.

**Table 5:** characteristics of thoracic drainage

Parameters (n=34)	Value (extremes)
Duration of drainage (median, day)	7 [3-16]
Number of instillation session of fibrinolysis (median)	4 [2-9]
Delay between drainage and first instillation of fibrinolysis (median, day)	1.6 [1-6]
Drained fluid volume after first instillation (median, ml)	800 [400-1500]
Total drained fluid volume (median, ml)	2450 [1400-4500]

**Table 6:** empirical antibiotic therapy

Probabilistic antibiotic therapy (n=34)	Number (%)
Amoxicillin-clavulanate	18 (52.94)
Amoxicillin	5 (14.7)
Amoxicillin + Metronidazole	4 (11.76)
Fluoroquinolones	2 (5.88)
Imipenem	2 (5.88)
Cephalosporin 3rd Generation + Fluoroquinolones	1 (2.94)
Vancomycin + Gentamycin	1 (2.94)
Oxacillin	1 (2.94)

### Complications assessment

CPPE recurrence was seen in 2 cases (5.9%). Complications included recurrent empyema had occurred in only one case after a month of ICU discharge. Surgery such as video-assisted thoracoscopic surgery (VATS) thoracotomy with pleural decortication, or other type of open surgical drainage were needed in 4 patients after medical treatment failure and there is evidence of significant pleural fibrosis. No major adverse effects (AE) were observed during application IPF application. The mean length of ICU stay was 15 days [6-31]. In one patient, CPPE was complicated with septic shock which leads to rapidly fatal multiple organ failure and death.

## Discussion

Our results concluded that IPF with streptokinase may be potentially effective for reducing the need for surgery in CPPE. IPF therapy is effective for shortening length of hospital stays without increasing the incidence of severe side effects. No major AE were described during application of the treatment protocol. Surgery was suggested in only 5 cases.

### Diagnosis

In the management of community-acquired pneumonia (CAP), recognition of an associated eventual PE should be always considered. In fact, Porcel *et al*. reported that this association could be observed in 20% of cases [4]. The positive diagnosis can be made easily using a TUS which may have two roles: identification and puncture localization. This exam was practice in all our patients. In this situation, pleural fluid analysis (i.e., pH, glucose, lactate dehydrogenase, bacterial cults) provides essential information to decide if it´s necessary to practice a CTD (in case of empyema). In our study, comorbidities were present in about half of cases (47%). In fact, CPPE and empyema are more common at extreme's age. At least two-thirds of patients will have an identifiable risk factor at presentation, which may include immunosuppression (most frequently human Immunodeficiency virus infection) diabetes mellitus, alcohol addiction and malnutrition [5]. Symptoms related to PEs depend on the volume and rate of fluid's accumulation, the degree of pleural inflammation and the underlying cardiopulmonary status. In our study, clinical features of pleural infection were mostly present in addition to clinical symptoms of pneumonia. Small effusions are usually asymptomatic, whereas moderate to large ones cause typically dyspnea which can progress to acute respiratory failure which has been observed in 41% of cases. An inflammatory syndrome was observed in all cases. Indeed, the presence of an increased inflammatory marker, such as C-reactive protein, an exudative liquid Rivalta positive, a hypoglycopleuria, over than 50% of altered neutrophils in the PE are highly suggestive of CPPE.

### Imaging findings

The most common imaging technique to identify CPPE is postero-anterior chest X-ray [6]. However, diagnosis confirmation is usually made by TUS. It should be easily accessible in these cases. The presence of septa on ultrasonography suggests CPPE and hyperechogenicity testified on the presence of pus in the pleural cavity [7]. In our study, the most common echographic appearance was hyperechoic in 59% of cases. It is also recommended that TUS be performed by the same physician who will do the puncture, in order to increase diagnostic yield and reduce eventual complications of thoracocentesis [8]. In addition, TUS is considered actually as a novel tool for prognostic stratifications of CAP in hospitalized patients in ICU [9]. In other situation, thoracic CT (which was performed in 19 patients) will provide more detailed information about fluid loculation in areas escaped by ultrasound. In our study, it showed an isolated PE in 15 cases and an associated pneumonia in 4 cases. This exam is particularly useful in distinguishing empyema with air-fluid levels from lung abscess [10].

### Treatment

There are many available options for the management of pleural fluid in patients with CPPE, ranging from prolonged antibiotic therapy and observation, to semi-invasive techniques such as therapeutic aspiration, CTD and intrapleural fibrinolytics, to invasive interventions such as thoracoscopy, thoracotomy or open drainage [11]. Bacteriological data´s pleural sepsis varies significantly between community acquired and nosocomial infections [12]. Early appropriate antibiotic therapy represents the major therapy for pneumonia and parapneumonic effusion. Initial antibiotic is generally dictated by treatment guidelines and secondary adjusted according to blood and pleural fluid microbial cultures. Empiric antibiotic must also cover anaerobic which is generally not easily identified in cultures [13]. In our study, the most prevalent organisms isolated were Streptococcus pneumoniae in 6 cases. Anaerobes have been found in 5 cases. Most common suggested empiric antibiotic regimen for community-acquired empyema is amoxicillin-clavulanate [14]. That's why this association has been started in 53% in our study. If the etiology of CPPE has been identified on the basis of bacterial cultures (blood or PF specimens) or rapid antigen tests for S. pneumoniae (urine or PF specimens), antimicrobial therapy should be redirected. Patients with nosocomial empyema necessitate adequate bacterial gram-negative coverage. Vancomycin may be added when methicillin-resistant infection is suspected.

When an infected pleural space progresses to fibrinopurulent phase, fibrin creates intrapleural locules that impede CTD. Intrapleural instillation of fibrinolytic drugs offers a theoretical benefit for lysing fibrin adhesions, promoting pleural drainage, and avoiding surgery. For this, it was used in our study when only CTD fails to improve clinical state of our patients. Few studies have reported the beneficial effects of therapy with streptokinase, urokinase, and tissue plasminogen activator (rtPA) to avoid surgery by improving the radiographic appearance of loculated effusions and promoting catheter drainage. Streptokinase is usually administered (like in our study) as 250,000 IU dilated in 100 to 200 mL saline daily for up to 7 days; urokinase is administered as 100,000 IU in 100 mL saline daily up to 3 days whereas rtPA is usually administered as 10 to 25 mg twice daily up to 3 days. Chest drains should be clamped for 2 to 8 hours following administration of the fibrinolytic. RtPA provides fibrinolytic activity by avoiding antigenicity of streptokinase.

Based on early reports of efficacy, the British Thoracic Society (BTS) [15] and the American College of Chest Physicians (ACCP) guidelines [16] recommend fibrinolytic drugs as first management options of CPPE. Therefore, observational data suggest that using fibrinolytic drugs in the intrapleural space can decrease the failure rate, reduce surgical referral, and improve chest tube drainage by cleaving intrapleural fibrinous septations [17]. In the study conducted by Misthos *et al*. [17], patients were randomized in two groups. The first group was managed with tube thoracostomy only; the second one was treated on combining tube thoracostomy and streptokinase instillation. The authors had demonstrated that thoracostomy was successful in 67.1% of cases in G1, whereas the installation of streptokinase led to a favorable outcome in 87.7% (p < 0.05) and significantly shortened length of ICU stay. Mortality rate, need to surgical interventions and length of hospital stay were also significantly lower in the fibrinolytic group.

In our study, we used instillation of streptokinase as a fibrinolytic; it was repeated as long as no serious complication occurred. Cameron *et al*. have shown that this process improves radiological progression and decreases means of drainage duration and hospital length of stay [18]. In the other side, safety profile of intrapleural fibrinolysis remains unclear. Dose and interval of fibrinolytic agents´ administration remain empiric and many considerations underlie wide variability in patient outcomes in trials of IPF therapy [19]. Our present study had some limitations. First, the total number of enrolled patients was limited. Second, when analyzing the need for surgical intervention as a primary outcome, we may need to consider the source of bias derived from using clinical judgment. The need for surgical intervention was based on individual clinical judgment. Decisions may vary among different physicians. Third, it is a non-comparative study which has asses only one group of patients treated initially with IPF (only by sterptokinase) without resorting surgery. Our results show that IPF with streptokinase may be potentially effective for reducing the need for surgery in CPPE. IPF therapy is effective for shortening length of hospital stays without increasing the incidence of severe side effects. In our study, therapeutic success rate was as expected, with a failure rate below those reported in literature. We present intrapleural instillation of fibrinolytics and VATS as part of the same protocol, in which fibrinolytic therapy is the first-line treatment.

## Conclusion

Our findings suggested that in CPPE and empyema, intrapleural fibrinolysis with streptokinase can certainly shorten hospitalization duration without increasing the incidence of severe side effects. Routine administration of IPF for management of CPPE or empyema may be especially indicated when the patient present contraindication for surgery or in case of unavailability of experienced thoracoscopist.

### What is known about this topic

- *Intrapleural fibrinolytic (IPF) therapy confers a benefit in reducing the requirement for surgical interventions;*
- *Limited evidence exists to guide clinicians in selecting the ideal drainage technique compatible with each situation because of the broad variation in intrapleural infection´s extent, presence of locules, comorbidities, respiratory status and underlying pathogen´s virulence;*
- *IPF with streptokinase can certainly shorten the length of hospital stay without increasing the incidence of severe side effects*.

### What this study adds

- *This is the largest Tunisian case series describing this technique which requires an important technical platform and trained operators;*
- *Our study highlights the importance of ultrasound in the follow-up in the treatment of pneumonic effusion and empyema;*
- *The tables in this paper are the most useful part bringing new data in the literature*.
